# Effect of short chain fatty acids on the expression of free fatty acid receptor 2 (*Ffar2*), *Ffar3* and early-stage adipogenesis

**DOI:** 10.1038/nutd.2014.25

**Published:** 2014-08-04

**Authors:** G Frost, Z Cai, M Raven, D T Otway, R Mushtaq, J D Johnston

**Affiliations:** 1 Nutrition and Dietetic Research Group, Investigative Medicine, Faculty of Medicine, Imperial College, London, UK; 2 Faculty of Health and Medical Sciences, University of Surrey, Surrey, UK

## Abstract

Adipose tissue has a major influence on insulin sensitivity. Stimulation of free fatty acid receptor 2 (FFAR2) has been proposed to influence adipocyte differentiation. We hypothesised that exposing preadipocytes to short chain fatty acids would induce earlier expression of nuclear receptors that co-ordinate adipogenesis, triglyceride accumulation and leptin secretion. 3T3-L1 preadipocytes were differentiated in the presence of 1 μM acetate, 0.1–10 μM propionate or vehicle control. In experiment 1, expression of *Ffar2* and nuclear receptor mRNA was measured by quantitative PCR over 48 h following onset of differentiation. In experiment 2, extracellular leptin concentration and intracellular triglyceride content were measured at days 0, 2, 4, 6, 8 and 10 following the onset of differentiation. Control cells exhibited similar temporal dynamics of gene expression, triglyceride accumulation and leptin secretion as reported previously. We were unable to detect expression of *Ffar3* mRNA at any stage of differentiation. Consistent with a lack of *Ffar2* expression in the first 24 h of differentiation, acetate and propionate had no significant effect on nuclear receptor expression. Furthermore, acetate or propionate treatment did not alter leptin concentration or triglyceride content. In conclusion, we observed no significant effect of propionate or acetate on adipogenesis in 3T3-L1 cells using validated quantitative techniques.

## Introduction

Adipose tissue has a major influence over the risk of developing type 2 diabetes. Its dysregulation is thought to lie at the centre of many metabolic abnormalities.^1^ The ability of preadipocytes to differentiate into mature adipocytes has a significant impact on insulin resistance.^2^ Limited numbers of adipocytes in a hypercaloric environment leads to hypertrophic morphology and ectopic lipid deposition in the liver, muscle and pancreas, which decreases insulin sensitivity. A better understanding of adipogenesis is therefore important for the maintenance of metabolic health.

Diets high in fermentable carbohydrate, which escapes digestion in the small intestine and is fermented to short-chain fatty acids (SCFA) in the colon, lead to a decrease in adiposity.^3,4^ In humans, diets high in fermentable carbohydrate also improve insulin-stimulated glucose uptake into adipocytes.^5^ The free fatty acid receptor 2 (FFAR2; previously called GPCR43) is a G protein-coupled receptor that has been shown to be activated by SCFA.^6^ More recently, free fatty acid release has been shown to be reduced by SCFA in wild type, but not in FFAR2 knockout mice, suggesting that FFAR2 expression is necessary for at least some of the biological effects of SCFA.^7^ We and the others have demonstrated that fermentable carbohydrates, which raise plasma SCFA, affect adipocyte size and function.^4,5^ Furthermore, work by Hong *et al.*^8^ has suggested that stimulation of FFAR2 with low doses of SCFA may increase adipocyte differentiation *in vitro*. If SCFA stimulate differentiation through the FFAR2 receptor, exposure of preadipocytes to SCFA may regulate molecular pathways considered to be key to cell differentiation.

We hypothesised that FFAR2 is expressed early after the induction of 3T3-L1 cell differentiation and that addition of SCFA influences the expression of nuclear receptors present during differentiation, affecting both the lipid content and the secretion of leptin. We tested this hypothesis using validated quantitative and qualitative techniques.

## Materials and methods

### Cell culture

3T3-L1 preadipocytes (ATCC, LGC Standards, Teddington, Middlesex, UK) were cultured in growth medium (Dulbecco's modified Eagle's medium (Invitrogen Ltd, Paisley, UK) supplemented with 10% fetal bovine serum (Invitrogen), antibiotic/antimycotic (Invitrogen) and sodium pyruvate (Sigma-Aldrich Co Ltd, Poole, Dorset, UK)). For experiments, 3T3-L1 preadipocytes were seeded into six-well plates. Two days postconfluence (day 0), growth medium was changed to differentiation medium (growth medium containing 0.5 mM IBMX (Sigma-Aldrich), 1 μM dexamethasone (Sigma-Aldrich) and 10 μg ml^−1^ insulin (Invitrogen)). In both experiments 1 and 2, cells were exposed to SCFA (acetate or propionate at the specified concentrations) or vehicle control during the entire differentiation process. A previous study reported effects of 0.1 μM acetate and propionate on markers of adipogenesis.^8^ Based on this study and our preliminary data of cAMP inhibition (not shown), we chose to use 1 μM as the SCFA concentration for our main experiments.

### Experiment 1

To characterise molecular changes during early differentiation events, cells were harvested in TRIzol (Invitrogen) at 0.5, 1, 2, 4, 8, 16, 24 and 48 h following the addition of the differentiation medium. Gene expression was measured by Taqman quantitative real-time PCR (qPCR). In this experiment, SCFA were used at 1 μM final concentration.

### Experiment 2

After 48 h in differentiation medium, cells were incubated in growth medium containing 10 μg ml^−1^ insulin. Forty-eight hours later, this was exchanged for normal growth medium, which was replaced every 2 days thereafter. At days 0, 2, 4, 6, 8 and 10, culture medium was collected for analysis of leptin concentration, and cell lysates were prepared for quantitative analysis of intracellular triglyceride content. For leptin and quantified intracellular triglyceride measurement, SCFA were used at a final concentration of 1 μM. The experimental protocol was then repeated for qualitative measurement of intracellular triglyceride content by oil red O stain. As acetate and propionate have been shown to be equally effective at stimulating adipogenesis in the past,^8^ propionate was used at 0.1–10 μM final concentration.

### qPCR

To explore the impact of SCFA on early stages of adipocyte differentiation, we chose nuclear receptors expressed early (neuron-derived orphan receptor 1 (NOR-1) and nerve growth factor-induced gene B (NGFI-B)), late (liver X receptor alpha (LXRα)) and throughout differentiation (glucocorticoid receptor (GR) and retinoid X receptor alpha (RXRα)) as defined previously,^9^ as well as the SCFA receptors FFAR2 and FFAR3 (previously called GPCR41). Total RNA was reverse transcribed using random primers and M-MLV Reverse Transcriptase (Promega Corporation, Southampton, UK). The resulting cDNA was then used for qPCR using ABsolute QPCR ROX Mix (Abgene, Epsom, UK), as described previously.^10^

### Leptin assays

Leptin concentration in culture medium was measured using Quantikine ELISA kits (R&D Systems Europe Ltd, Abingdon, UK)^10^ and normalised to total cellular protein, measured using a Pierce BCA Protein Assay Kit (Thermo Fisher Scientific, Cramlington, UK).

### Quantitative triglyceride analysis

Cells were rinsed twice with 1 × phosphate-buffered saline, scraped into 0.1 M sodium dodecyl sulfate solution in 1 × phosphate-buffered saline and lysed by vortex mixing and sonication. Triglyceride was measured using an Alfa Wassermann triglyceride assay (Randox Laboratories Ltd, Crumlin, UK). Triglyceride content was normalised to total cellular protein and measured using a Pierce BCA Protein Assay Kit (Thermo Fisher Scientific).^10^

### Oil red O stain

Cells were stained for qualitative assessment of triglyceride content as described previously.^10^

### Statistical analysis

Quantitative gene expression data represent the mean±s.e.m. of *n*=4 groups of cells from a representative experiment. Intracellular triglyceride and secreted leptin data are pooled from *n*=4 separate experiments. These data were analysed by two-way analysis of variance (ANOVA) with Bonferoni's *post hoc* analysis.

## Results

**Figure 1:**
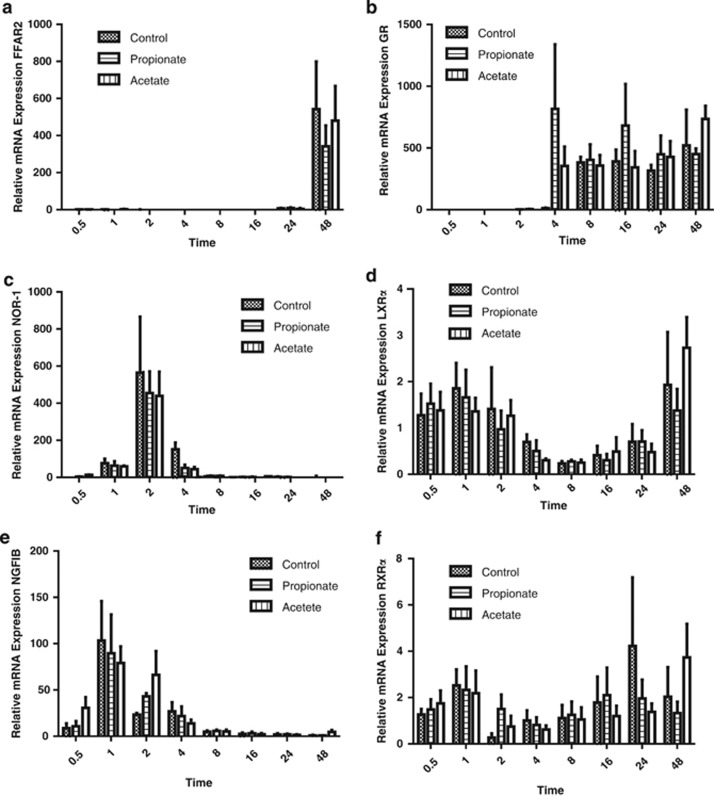
*Ffar2* and nuclear receptor mRNA expression during 3T3-L1 cell adipogenesis. The expression of *Ffar2* and nuclear factors at 0.5, 1, 2, 4, 8, 16, 24 and 48 h following the induction of differentiation in 3T3-L1 preadipocytes with acetate (vertical lines), propionate (horizontal lines) or vehicle control (hatched bars) added to the media. There was no significant (*P*>0.05, two-way ANOVA) effect of treatment or time × treatment interaction for any of the genes measured.

Expression of *Ffar2* was only observed 48 h after the onset of differentiation (Figure 1a). Overall, there was a significant (*P*<0.001, two-way ANOVA) effect of time on *Ffar2* expression but no significant (*P*>0.05, two-way ANOVA) effect of treatment or time × treatment interaction. We could not detect *Ffar3* mRNA at any stage of adipogenesis in any of the treatment groups. There was no significant (*P*>0.05, two-way ANOVA) effect of time, treatment or time × treatment interaction on the expression of *Rxrα* (Figure 1f). For all other nuclear receptor genes, there was a significant (*P*<0.001, two-way ANOVA) effect of time on expression but no significant (*P*>0.05, two-way ANOVA) effect of treatment or time × treatment interaction.

**Figure 2:**
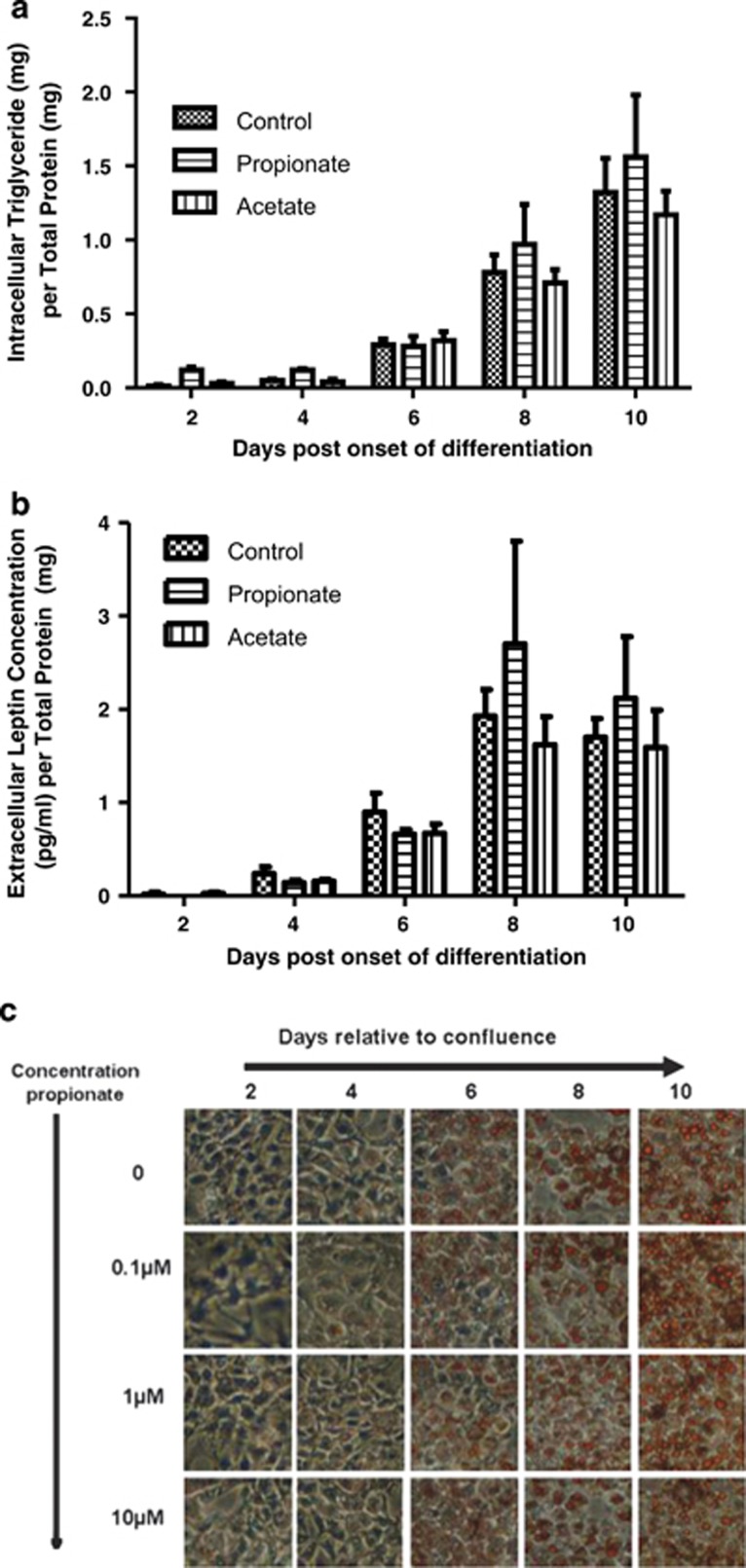
Triglyceride accumulation and leptin secretion during 3T3-L1 cell adipogenesis. (**a**) The lipid content and (**b**) leptin output of 3T3-L1 cells following induction of differentiation of 3T3-L1 preadipocytes at days 0, 2, 4, 6, 8 and 10 with acetate (horizontal lines), propionate (white) or control (black) added to the media. There was no significant (*P*>0.05, two-way ANOVA) effect of treatment or time × treatment interaction for either of the parameters measured. (**c**) Oil red O staining following induction of differentiation of 3T3-L1 preadipocytes at days 0, 2, 4, 6, 8 and 10 with propionate.

There was a significant (*P*<0.001, two-way ANOVA) effect of time on intracellular triglyceride content (Figure 2a) and extracellular leptin concentration (Figure 2b). However, there was no significant (*P*>0.05, two-way ANOVA) effect of treatment or time × treatment interaction on either of these factors. Oil red O staining allowed further assessment of intracellular triglyceride content and qualitative analysis of cellular morphology. Because of the lack of effect of acetate and propionate above, we used a range of concentrations, from 0.1 to 10 μM. However, none of the treatment groups exhibited a detectable change in oil red O staining or cell morphology with propionate (Figure 2c).

## Discussion

Insulin sensitivity deteriorates with increased ectopic fat deposition in the liver, muscle and pancreas.^2^ This may be due to limited numbers of hypertrophic adipocytes unable to store the lipid effectively in a hypercaloric environment.^2^ Understanding factors that effect adipocyte differentiation may therefore improve metabolic flexibility.

Recent evidence has demonstrated that stimulation of FFAR2 with the SCFA acetate and propionate causes a reduction in free fatty acid release from the adipocyte, and this effect is lost in tissue from the *Ffar2* knockout mouse.^7^ Using 3T3-L1 cells, it has been suggested that exposure of adipocytes to low concentrations of acetate and propionate increases differentiation and intracellular lipid content.^8^ Our initial aim was to extend this work by testing the hypothesis that SCFA would influence the expression of nuclear receptors present during differentiation.

Here we have shown in 3T3-L1 cells that *Ffar2* mRNA is not expressed until approximately 48 h into the differentiation process. Exposing preadipocytes to either propionate or acetate from the induction of differentiation made no difference to the time to expression of *Ffar2* mRNA over the time course studied. We were unable to detect *Ffar3*, consistent with other published data.^8^ We measured mRNA of five nuclear receptors that are implicated in adipogenesis and reported to be expressed early (NOR-1 and NGFI-B), late (LXRα) and throughout differentiation (RXRα and GR).^9^ Control groups exhibited similar patterns of expression of these genes over 48 h as previously reported.^9^ However, consistent with the lack of effect of acetate or propionate on the timing of onset of *Ffar2* expression, we were unable to demonstrate an effect of SCFA on nuclear receptor expression at any stage within the initial 48 h of differentiation. These data imply that at low physiological concentrations SCFA are unable to regulate key molecular events of adipogenesis without the expression of *Ffar2* or *Ffar3*.

Given the lack of effect on established molecular markers of early adipogenesis, we then examined the effects of SCFA on physiological changes. In contrast to the data of Hong *et al.*,^8^ using a 10 times higher concentration, we failed to observe a significant effect of SCFA on intracellular triglyceride content and leptin secretion occurring after the onset of *Ffar2* mRNA expression. Moreover, using a range of 0.1–10 μM propionate, we were unable to observe any changes in oil red O staining or gross cellular morphology.

In conclusion, our data do not support a major role for SCFA in adipogenesis of 3T3-L1 cells.
